# Hygro-Thermal Vibrations of Porous FG Nano-Beams Based on Local/Nonlocal Stress Gradient Theory of Elasticity

**DOI:** 10.3390/nano11040910

**Published:** 2021-04-02

**Authors:** Rosa Penna, Luciano Feo, Giuseppe Lovisi, Francesco Fabbrocino

**Affiliations:** 1 Department of Civil Engineering, University of Salerno, 84084 Fisciano, Italy; lfeo@unisa.it (L.F.); glovisi@unisa.it (G.L.); 2 Department of Engineering, Pegaso Telematic University, 80143 Naples, Italy; francesco.fabbrocino@unipegaso.it

**Keywords:** porous functionally graded materials, nano-beams, dynamics, local/nonlocal stress gradient elasticity, hygro-thermal loadings

## Abstract

In this manuscript the dynamic response of porous functionally-graded (FG) Bernoulli–Euler nano-beams subjected to hygro-thermal environments is investigated by the local/nonlocal stress gradient theory of elasticity. In particular, the influence of several parameters on both the thermo-elastic material properties and the structural response of the FG nano-beams, such as material gradient index, porosity volume fraction, nonlocal parameter, gradient length parameter, and mixture parameter is examined. It is shown how the proposed approach is able to capture the dynamic behavior of porous functionally graded Bernoulli–Euler nano-beams under hygro-thermal loads and leads to well-posed structural problems of nano-mechanics.

## 1. Introduction

In the last decades, nanostructures have become a subject of great interest among academic researchers due to their wide of application potentials including high-tech devices in nano-scaled systems such as NEMS (nano electromechanical systems) [1,2,3,4,5]. Nowadays, with the rapidly development of the fabrication technology of materials, the concept of functionally graded materials (FGMs) is more and more extended to nanotechnologies in order to design nano-scaled structures for use in the most extreme conditions, including extremely high ambient temperatures and absorbed moisture environments [6,7,8,9,10,11]. In this way, the use of temperatures-dependent FGMs allows to improve the overall performance of nanostructures as well as to ensure their structural integrity when they are exposed to the coupled effect of mechanical loads and hygro-thermal environments. So that, it is necessary to study in depth their response in such loading conditions.

As demonstrated by the results of experimental nanoscale tests and atomistic simulations [12,13], nanostructures exhibit a size-dependent structural response that can be captured by resorting the non-classical continuum models including both nonlocal theories of elasticity and nonlocal gradient ones. These theories are widely applied to capture the nano-scale effects, but in a different way: the first one is a formulation based on a single length scale parameter, while the second one is based on two length scale parameters in order to consider both of the microstructure strain mechanism and the inter-atomic long-range force. In addition, nonlocal theories are able to predict only softening or hardening material response as opposed to nonlocal gradient ones capable to predict both softening and hardening behaviors of the material at nano-scale. In the framework of nonlocal elasticity, two of the most notable purely nonlocal constitutive laws are surely the softening or Eringen’s strain-driven nonlocal integral model (StrainDM) [14,15], in which the total stress of a given point is a function of the strain at all other adjacent points of the continuum, and the more recently hardening or stress-driven nonlocal integral model (StressDM) developed by Romano and Barretta [16], in which the strain at any point is resulted from the stress of all points. As widely discussed in [17,18,19], the differential formulation of StrainDM is ill-posed and leads to the unexpected paradoxical results for some boundary and loading conditions, unlike the well-posed StressDM that provides a consistent approach for the analysis of nanostructures [20,21,22,23,24,25,26,27,28,29,30,31].

In addition, Lim et al. [32] introduced the nonlocal strain gradient theory (Lim’s NStrainGT) in order to generalize the Eringen’s nonlocal model by combining it with the strain gradient model [33,34] in which the total stress is a function of the strain and its gradient not only at the reference point x, but also at all other points within the domain. Although this model is extensively applied for many years by several researchers in a large number of investigations, recently Zaera et al. [35] have declared that the nonlocal strain gradient theory leads to ill-posed structural problems since the constitutive boundary conditions are in conflict with both non-standard kinematic and static higher-order boundary conditions.

The ill-posed problem related to the Lim’s NStrainGT model may be bypassed by resorting to the Eringen local-nonlocal mixture constitutive model [36] or by using coupled theories based on the combination of pure nonlocal theory with the surface theory of elasticity [37]. In the last years, some applications of the theories mentioned above are addressed in [38,39,40,41] and [42,43], respectively.

The ill-posedness of Lim’s NStrainGT can be advantageously circumvented using the variationally consistent nonlocal gradient formulations, such as local/nonlocal strain-driven gradient (L/NStrainG) and local/nonlocal stress-driven gradient (L/NStressG) theories, conceived by Barretta et al. in [44,45] for both static and dynamics problems. These novel constitutive formulations lead to well-posed structural problems of nano-mechanics.

The motivation of the present paper is to extend the analysis on the hygro-thermal bending behavior of porous FG nano-beams, developed in [46] by using the aforementioned consistent nonlocal gradient formulations, to their dynamic response, against of many articles on the topic in which the hygro-thermal effects on the size-dependent behavior of nanostructures have been analyzed by making recourse to Eringen’s nonlocal model [47,48,49,50,51,52,53] or Lim’s nonlocal strain gradient theory in [54,55,56], more popularities due to their simply differential formulation.

The main assumptions and simplifications used for studying the nonlocal vibration characteristics of porous functionally graded within hygro-thermal environments are the following:

- a slender and perfectly straight porous FG nano-beam of an Euler–Bernoulli type, with rectangular cross-section, is considered; hence, the influence of thickness stretching and shear deformation are neglected;
- the material properties are assumed to be temperature dependent following a nonlinear equation and to vary continuously through the thickness according to a power law distribution in terms of the volume fractions of the constituents;
- both a uniform temperature variation and a moisture concentration are assumed to occur in the thickness direction only;
- the thermo-elastic material properties are evaluated with respect to the elastic center of the nano-beam cross-section; hence, the bending–extension coupling due to the variation of the functionally graded material is eliminated;
- the influence of a temperature-dependent rotary inertia has been considered.

The paper is structured as follows: The effective mechanical and hygro-thermal properties of the FG material, as well as the equations of motion of the porous Bernoulli–Euler nano-beams are derived in Section 2 by using the Hamilton’s principle. In Section 3, the local/nonlocal stress-driven gradient model of elasticity is introduced. In Section 4 the dimensionless governing equations of the linear transverse free vibrations are obtained. The solution procedure is presented in Section 5 and validated in Section 6 where the main results of the free vibration analysis are also presented and discussed. Some closing remarks are provided in Section 7.

## 2. Problem Formulation

### 2.1. Temperature-Dependent Porous FG Nano-Beam 

**Figure 1:**
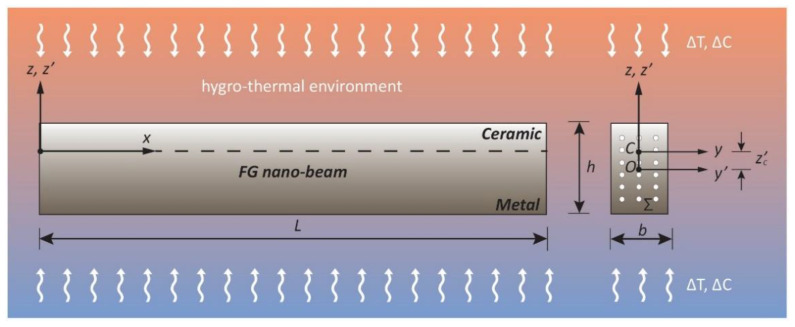
Coordinate system and configuration of a porous FG Bernoulli–Euler nano-beam.

Let us consider a Bernoulli–Euler nano-beam made of a metal-ceramic functionally graded (FG) porous material with length “*L*”, thickness “*h*” and width “*b*”, undergoing hygro-thermal loads (Figure 1). 

By denoting by *y’* and *z’* the principal axes of geometric inertia originating at the geometric center *O* of the nano-beam rectangular cross-section, *Σ*(*x*), the effective value of the FG material generic property, f(z’), including the mass density, ρ(*z’*), the Young’s modulus, *E*(*z’*), the thermal expansion coefficient, *α*(*z’*), and the moisture expansion coefficient, *β*(*z’*), can be evaluated by the following rule of mixture equation:

(1) $$f\left(z’\right)=f_{m}+\left(f_{c}-f_{m}\right){\left(\frac{1}{2}+\frac{z^{’}}{h}\right)}^{k}-\frac{\zeta }{2}\left(f_{c}+f_{m}\right)$$

being $f_{c}$ and $f_{m}$ the generic thermo-elastic and physical properties of ceramic (Si_3_N_4_) and metal (SuS_3_O_4_), whose characteristic values$,\;P_{0},$ are listed in Table 1 [46]; *k* (*k* ≥ 0) and *ζ* (*ζ* << 1) are the gradient index and the porosity volume fraction of the FG material, respectively. 

Moreover, the following nonlinear equation is introduced to express the temperature dependence of the thermo-elastic property, P(T):

(2) $$P\left(T\right)=P_{0}\left(1+X_{-1}\;T^{-1}+X_{1}\;T+X_{2}\;T^{2}+X_{3}\;T^{3}\right)$$

where $X_{-1},X_{1},X_{2}\;$and $X_{3}\;$are the coefficients of the two constituent materials (Table 2).

In this investigation, both a uniform temperature rise, $T\left(z^{’}\right)=T_{b}+\Delta T,\;$and a moisture concentration$,\;C\left(z^{’}\right)=C_{b}+\;\Delta C,\;$between the bottom (*z’* = −h/2) and the top surface (z’ = +h/2) of the nano-beam cross-section are considered (Figure 1), being $T\left(z^{’}\right)\;$and $C\left(z^{’}\right)$ the current values of the temperature and moisture through the thickness direction (*z’*), $T_{b}=305\;\left[K\right]$ and $C_{b}=0\;\left[{\mathrm{wt}\%H}_{2}O\right]\;$the reference values of the temperature and moisture concentration at the bottom surface, and ΔT, ΔC the increments of the temperature and moisture concentration, respectively.

In order to eliminate the bending–extension coupling due to the variation of the functionally graded material, the thermo-elastic material properties are evaluated with respect to a new elastic Cartesian coordinate system originating at the elastic center *C*, whose position, $z_{c}^{’},\;$is given as:

(3) $$z_{c}^{’}=\frac{\int_{\Sigma }E\left(z^{’},T\right)z^{’}d\Sigma \;}{\int_{\Sigma }E\left(z^{’},T\right)d\Sigma \;}$$

Therefore, the coordinate z originating at C is given by z = z’–$z_{c}^{’\;},\;$while y = y’.

### 2.2. Governing Equation

Based on Bernoulli–Euler theory, the displacement field components $\left(u_{x},\;u_{z}\right)$ and the corresponding nonzero strain ($\varepsilon_{xx}$) are expressed as follows:

(4) $$u_{x}\left(x,z,t\right)=u\left(x,t\right)-z\frac{\partial w}{\partial x}\left(x,t\right)$$

(5) $$u_{z}\left(x,z,t\right)=w\left(x,t\right)$$

(6) $$\varepsilon_{xx}\left(x,z,t\right)=\;\frac{\partial u\left(x,t\right)}{\partial x}-\;z\frac{\partial^{2}w\left(x,t\right)}{\partial x^{2}}$$

where *u* (*x*,*t*) and *w* (*x*,*t*) are the axial and transverse displacements of the elastic centre *C*, at time *t*, respectively, and the term $\frac{\partial^{2}w\left(x,t\right)}{\partial x^{2}}$ refers to the geometrical curvature χ.

Now, by using Hamilton’s principle [49], the equations of motion can be derived as:

(7) $$\frac{\partial N\left(x,t\right)}{\partial x}=A_{\rho }\frac{\partial^{2}u\left(x,t\right)}{\partial t^{2}}\;,$$

(8) $$\frac{\partial^{2}M\left(x,t\right)}{\partial x^{2}}-\left(N^{T}+N^{C}\right)\frac{\partial^{2}w\left(x,t\right)}{\partial x^{2}}=A_{\rho }\frac{\partial^{2}w\left(x,t\right)}{\partial t^{2}}-I_{\rho }\frac{\partial^{4}w\left(x,t\right)}{\partial x^{2}\;\partial t^{2}},$$

with the corresponding boundary conditions at *x* = [*0*,*L*]:

(9) u(x,t)   or   N(x,t),

(10) $$-\;\frac{\partial w\left(x,t\right)}{\partial x}\;\mathrm{or}\;\;\;M\left(x,t\right),$$

(11) $$w\left(x,t\right)\;\;\mathrm{or}\;\;\;\;V\left(x,t\right)=\frac{\partial M\left(x,t\right)}{\partial x}-\left(N^{T}+N^{C}\right)\frac{\partial w\left(x,t\right)}{\partial x},$$

where N(x,t), M(x,t), and V(x,t) denote the local axial force, the bending moment resultant and the equivalent shear force, respectively. In Equations (7) and (8), $I_{\rho }$ and $A_{\rho }$ are, respectively, the temperature-dependent rotary inertia and the effective cross-sectional mass of the porous FG nano-beam, expressed as follow:

(12) $$I_{\rho }=b\int_{-\frac{h}{2}-z_{c}^{’}}^{\frac{h}{2}-z_{c}^{’}}\rho \left(z,T\right)z^{2}dz,$$

(13) $$A_{\rho }=b\int_{-\frac{h}{2}-z_{c}^{’}}^{\frac{h}{2}-z_{c}^{’}}\rho \left(z,T\right)dz,$$

and $N^{T}\;$and $N^{C}\;$denote the hygro-thermal axial force resultants, defined as:

(14) $$N^{T}=N^{T}\left(z,T\right)=\;\int_{\Sigma }E\alpha \Delta Tdz,$$

(15) $$N^{C}=N^{C}\left(z,T\right)=\;\int_{\Sigma }E\beta \Delta Cdz,$$

in which α=α(z,T) and β=β(z,T) are the thermal and moisture expansion temperature-dependent coefficients, respectively, previously defined, and E=E(z,T).

## 3. Local/Nonlocal Stress Gradient Formulation

By using the local/nonlocal stress gradient integral formulation [46], the elastic axial strain component,${\;\varepsilon }_{xx}^{el}$, is expressed by the following constitutive mixture equation:

(16) $$\varepsilon_{xx}^{el}\;=\xi_{1}\frac{\sigma_{xx}\left(x,t\right)}{E}+\frac{1-\xi_{1}}{E}\;\int_{0}^{L}\Phi_{L_{c}}\left(x-\xi \right)\;\sigma_{xx}\left(\xi ,t\right)d\xi -\frac{1}{E}L_{l}^{2}\;\frac{\partial }{\partial x}\int_{0}^{L}\Phi_{L_{c}}\left(x-\xi \right)\;\frac{\partial \sigma_{xx}\left(\xi ,t\right)}{\partial x}d\xi ,$$

where x and ξ are the position vectors of the points of the domain at time *t*; $\sigma_{xx}\;$and $\frac{\partial \sigma_{xx}\;}{\partial_{x}}$ denote the axial stress component and its gradient, respectively;$\;\xi_{1}$ is the mixture parameter and $L_{l}$ is the gradient length parameter. 

Moreover, $\Phi_{L_{c}}$, is the bi-exponential function of the scalar averaging kernel:

(17) $$\Phi_{L_{c}}\left(x,\;L_{c}\right)=\frac{1}{2L_{c}}\exp\;(-\;\frac{\left|x\right|}{L_{c}}\;),$$

depending on the length-scale parameter$,L_{c},$ which describe the nonlocal effects. 

In hygro-thermal environment, the elastic axial strain is expressed by the following relation:

(18) $$\varepsilon_{xx}^{el}=\varepsilon_{xx}-\varepsilon_{xx}^{*},$$

in which,$\;\varepsilon_{xx}$, is the total axial strain and$,{\;\varepsilon }_{xx}^{*}$ = α ΔT+βΔC, denotes the non-elastic axial strain depending on the increases in temperature, ΔT, and moisture concentration, ΔC.

As it is well-known, by choosing a smoothing function as Equation (17), the integro-differential Equation (16) can be rewritten in the following differential form:

(19) $$\varepsilon_{xx}^{el}-L_{c}^{2}\;\frac{\partial^{2}\varepsilon_{xx}^{el}}{\partial x^{2}}=\frac{\sigma_{xx}}{E}-\frac{L_{c}^{2}}{E}\left(\xi_{1}+\frac{L_{l}^{2}}{L_{c}^{2}}\right)\frac{\;\partial^{2}\sigma_{xx}}{\partial x^{2}},$$

equipped with the constitutive boundary conditions (CBCs) at the nano-beam ends:

(20) $$\frac{\partial \varepsilon_{xx\;}^{el}\left(0,t\right)}{\partial x}-\frac{1}{L_{c}}\varepsilon_{xx}^{el}\left(0,t\right)=\;-\frac{1}{E}\frac{\xi_{1}}{L_{c}}\sigma_{xx}\left(0,t\right)+\frac{1}{E}\left(\xi_{1}+\frac{L_{l}^{2}}{L_{c}^{2}}\right)\;\frac{\partial \sigma_{xx}\left(0,t\right)}{\partial x},\;$$

(21) $$\frac{\partial \varepsilon_{xx}^{el}\;\left(L,t\right)}{\partial x}+\frac{1}{L_{c}}\varepsilon_{xx}^{el}\left(L,t\right)=\;\frac{1}{E}\frac{\xi_{1}}{L_{c}}\sigma_{xx}\left(L,t\right)+\frac{1}{E}\left(\xi_{1}+\frac{L_{l}^{2}}{L_{c}^{2}}\right)\;\frac{\partial \sigma_{xx}\left(L,t\right)}{\partial x}.$$

Next, by substituting Equation (6) into Equations (19)–(21), then multiplying by (1, z), the integration over the nano-beam cross section provides the following NStressG equations in terms of axial and transverse displacement:

(22) $$\frac{\partial u\left(x,t\right)}{\partial x}-L_{c}^{2}\;\frac{\partial^{3}u\left(x,t\right)}{\partial x^{3}}-\frac{\left(N^{T}+N^{C}\right)}{A_{E}}=\frac{N^{NStressG}\left(x,t\right)}{A_{E}}-\;\frac{L_{c}^{2}}{A_{E}}\left(\xi_{1}+\frac{L_{l}^{2}}{L_{c}^{2}}\right)\frac{\;\partial^{2}N^{NStressG}\left(x,t\right)}{\partial x^{2}},$$

(23) $$-\frac{\partial^{2}w\left(x,t\right)}{\partial x^{2}}+L_{c}^{2}\;\frac{\partial^{4}w\left(x,t\right)}{\partial x^{4}}=\frac{M^{NStressG}\left(x,t\right)}{I_{E}}-\frac{L_{c}^{2}}{I_{E}}\left(\xi_{1}+\frac{L_{l}^{2}}{L_{c}^{2}}\right)\frac{\partial^{2}M^{NStressG}\left(x,t\right)}{\partial x^{2}},$$

with two pairs of CBCs:

(24) $$\frac{\partial^{2}u\left(0,t\right)}{\partial x^{2}}-\frac{1}{L_{c}}\frac{\partial u\left(0,t\right)}{\partial x}=\;-\frac{1}{A_{E}}\frac{\xi_{1}}{L_{c}}N^{NstressG}\left(0,t\right)+\frac{1}{A_{E}}\left(\xi_{1}+\frac{L_{l}^{2}}{L_{c}^{2}}\right)\;\frac{\partial N^{NStressG}\left(0,t\right)}{\partial x},\;$$

(25) $$\frac{\partial^{2}u\left(L,t\right)}{\partial x^{2}}+\frac{1}{L_{c}}\frac{\partial u\left(L,t\right)}{\partial x}=\;\frac{1}{A_{E}}\frac{\xi_{1}}{L_{c}}N^{NstressG}\left(L,t\right)+\frac{1}{A_{E}}\left(\xi_{1}+\frac{L_{l}^{2}}{L_{c}^{2}}\right)\frac{\partial N^{NStressG}\left(L,t\right)}{\partial x}\;,$$

(26) $$-\frac{\partial^{3}w}{\partial x^{3}}\left(0,t\right)+\frac{1}{L_{c}}\frac{\partial^{2}w}{\partial x^{2}}\left(0,t\right)=\;-\frac{1}{I_{E}}\frac{\xi_{1}}{L_{c}}M^{NstressG}\left(0,t\right)+\frac{1}{I_{E}}\left(\xi_{1}+\frac{L_{l}^{2}}{L_{c}^{2}}\right)\frac{\partial M^{NStressG}\left(0,t\right)}{\partial x},$$

(27) $$-\frac{\partial^{3}w}{\partial x^{3}}\left(L,t\right)-\frac{1}{L_{c}}\frac{\partial^{2}w}{\partial x^{2}}\left(L,t\right)=\frac{1}{I_{E}}\frac{\xi_{1}}{L_{c}}M^{NstressG}\left(L,t\right)+\frac{1}{I_{E}}\left(\xi_{1}+\frac{L_{l}^{2}}{L_{c}^{2}}\right)\frac{\partial M^{NStressG}\left(L,t\right)}{\partial x},$$

in which $N^{NStressG}$ and $M^{NStressG}$ denote the local/nonlocal stress gradient axial force and moment resultants; $A_{E}$ and $I_{E}$ are, respectively, the axial and bending stiffnesses of a FG nano-beam, defined as:

(28) $$I_{E}=b\int_{-\frac{h}{2}-z_{c}^{’}}^{\frac{h}{2}-z_{c}^{’}}E\left(z,T\right)z^{2}dz,$$

(29) $$A_{E}=b\int_{-\frac{h}{2}-z_{c}^{’}}^{\frac{h}{2}-z_{c}^{’}}E\left(z,T\right)dz.$$

Furthermore, by substituting Equations (7) and (8) into Equations (22) and (23), the local/nonlocal stress gradient axial force and moment resultants can be described explicitly in terms of displacement components as follows:

(30) $$N^{NStressG}\left(x,t\right)=\;A_{E}\left(\frac{\partial u\left(x,t\right)}{\partial x}-L_{c}^{2}\frac{\partial^{3}u\left(x,t\right)}{\partial x^{3}}\right)-\left(N^{T}+N^{C}\right)+L_{c}^{2}\left(\xi_{1}+\frac{L_{l}^{2}}{L_{c}^{2}}\right)A_{\rho }\frac{\partial^{3}u\left(x,t\right)}{\partial x\partial t^{2}},$$

(31) $$M^{NStressG}\left(x,t\right)=-I_{E}\frac{\partial^{2}w\left(x,t\right)}{\partial x^{2}}+I_{E}L_{c}^{2}\;\frac{\partial^{4}w\left(x,t\right)}{\partial x^{4}}+L_{c}^{2}\left(\xi_{1}+\frac{L_{l}^{2}}{L_{c}^{2}}\right)\left(A_{\rho }\frac{\partial^{2}w\left(x,t\right)}{\partial t^{2}}-I_{\rho }\frac{\partial^{4}w\left(x,t\right)}{\partial x^{2}\partial t^{2}}+\left(N^{T}+N^{C}\right)\frac{\partial^{2}w\left(x,t\right)}{\partial x^{2}}\right).$$

Finally, by employing Equations (7), (8), (30), and (31) the following local/nonlocal stress gradient equations of motion are derived:

(32) $$A_{E}\frac{\partial^{2}u\left(x,t\right)}{\partial x^{2}}-L_{c}^{2}A_{E}\frac{\partial^{4}u\left(x,t\right)}{\partial x^{4}}-\frac{\partial }{\partial x}\left(N^{T}+N^{C}\right)=A_{\rho }\frac{\partial^{2}u\left(x,t\right)}{\partial t^{2}}-L_{c}^{2}\left(\xi_{1}+\frac{L_{l}^{2}}{L_{c}^{2}}\right)A_{\rho }\frac{\partial^{4}u\left(x,t\right)}{\partial x^{2}\partial t^{2}},$$

(33) $$-I_{E}\;\frac{\partial^{4}w\left(x,t\right)}{\partial x^{4}}+I_{E}L_{c}^{2}\frac{\partial^{6}w\left(x,t\right)}{\partial x^{6}}+L_{c}^{2}\left(\xi_{1}+\frac{L_{l}^{2}}{L_{c}^{2}}\right)\frac{\partial^{2}}{\partial x^{2}}\left(A_{\rho }\frac{\partial^{2}w\left(x,t\right)}{\partial t^{2}}-I_{\rho }\frac{\partial^{4}w\left(x,t\right)}{\partial x^{2}\;\partial t^{2}}+\left(N^{T}+N^{C}\right)\frac{\partial^{2}w\left(x,t\right)}{\partial x^{2}}\right)=\;\;\left(A_{\rho }\frac{\partial^{2}w\left(x,t\right)}{\partial t^{2}}-I_{\rho }\;\frac{\partial^{4}w\left(x,t\right)}{\partial x^{2}\;\partial t^{2}}+\left(N^{T}+N^{C}\right)\frac{\partial^{2}w\left(x,t\right)}{\partial x^{2}}\right),$$

with the following natural boundary conditions at the nano-beam ends (x=0,L):

(34) $$N^{NstressG}\left(x,t\right)=\;\bar{N},$$

(35) $$\frac{\partial M^{NstressG}\left(x,t\right)}{\partial x}-\left(N^{T}+N^{C}\right)\frac{\partial w\left(x,t\right)}{\partial x}\;=\bar{V},$$

(36) $$M^{NstressG}\left(x,t\right)=\bar{M},$$

being $\bar{N}$, $\bar{M}$, and$\;\bar{V}$ the assigned generalized forces acting at the nano-beam ends together and with the aforementioned CBCs at the nano-beam ends given by Equations (24)–(27).

## 4. Linear Transverse Free Vibration Analysis

Firstly, the following dimensionless quantities are introduced:

$$\frac{x}{L}=\tilde{x},$$

$$\frac{w\left(x,t\right)}{L}=\tilde{w}{\left(\tilde{x},t\right)}_{,}$$

$$\frac{L_{c}}{L}=\lambda_{c},$$

$$\frac{L_{l}}{L}=\lambda_{l},$$

$$\frac{N^{T}}{I_{E}}L^{2}=\tilde{N^{T}},$$

$$\frac{N^{C}}{I_{E}}L^{2}=\tilde{N^{C}},$$

$$\frac{A_{\rho }L^{4}}{I_{E}}=\tilde{A_{\rho }},$$

$$\frac{1}{L^{2}}\frac{I_{\rho }}{\tilde{A_{\rho }}}=\tilde{g^{2}},$$

(37) $$\omega^{2}\tilde{A_{\rho }}={\tilde{\omega }}^{2}.$$

Applying these quantities, the dimensionless governing equations of the linear transverse free vibrations associated with NStressG constitutive formulation can be obtained as follows:

- Dimensionless free vibration equation

(38) $$\lambda_{c}^{2}\frac{\partial^{6}\tilde{w}\left(\tilde{x},t\right)}{\partial {\tilde{x}}^{6}}-\frac{\partial^{4}\tilde{w}\left(\tilde{x},t\right)}{\partial {\tilde{x}}^{4}}+\lambda_{c}^{2}\left(\xi_{1}+\frac{\lambda_{l}^{2}}{\lambda_{c}^{2}}\right)\left(\tilde{N^{T}}+\tilde{N^{C}}\right)\frac{\partial^{4}\tilde{w}\left(\tilde{x},t\right)}{\partial {\tilde{x}}^{4}}-\left(\tilde{N^{T}}+\tilde{N^{C}}\right)\frac{\partial^{2}\tilde{w}\left(\tilde{x},t\right)}{{\tilde{x}}^{2}}\;=\tilde{A_{\rho }}\left(\frac{\partial^{2}\tilde{w}\left(\tilde{x},t\right)}{\partial t^{2}}-\tilde{g^{2}}\frac{\partial^{4}\tilde{w}\left(\tilde{x},t\right)}{\partial {\tilde{x}}^{2}\partial t^{2}}\right)-{\tilde{\omega }}^{2}\lambda_{c}^{2}\left(\xi_{1}+\frac{\lambda_{l}^{2}}{\lambda_{c}^{2}}\right)\left(\frac{\partial^{4}\tilde{w}\left(\tilde{x},t\right)}{\partial {\tilde{x}}^{2}\partial t^{2}}-\tilde{g^{2}}\frac{\partial^{6}\tilde{w}\left(\tilde{x},t\right)}{\partial {\tilde{x}}^{4}\partial t^{2}}\right),$$

- Dimensionless standard boundary conditions

(39) $$\tilde{w}\left(\tilde{x},t\right)=\tilde{w^{*}},\;\mathrm{or}\;\frac{\partial {\tilde{M}}^{NStressG}\left(\tilde{x},t\right)}{\partial \tilde{x}}-\left(\tilde{N^{T}}+\tilde{N^{C}}\right)\frac{\partial \tilde{w}\left(\tilde{x},t\right)}{\partial \tilde{x}}=\tilde{V},$$

(40) $$-\frac{\partial \tilde{w}\left(\tilde{x},t\right)}{\partial \tilde{x}}=\frac{\partial {\tilde{w}}^{*}}{\partial \tilde{x}},\;\mathrm{or}\;{\tilde{M}}^{NStressG}\left(\tilde{x},t\right)=\tilde{M},$$

- Dimensionless constitutive boundary conditions

(41) $$-\frac{\partial^{3}\tilde{w}\left(0,t\right)}{\partial {\tilde{x}}^{3}}+\frac{1}{\lambda_{c}}\frac{\partial^{2}\tilde{w}\left(0,t\right)}{\partial {\tilde{x}}^{2}}=-\frac{\xi_{1}}{\lambda_{c}}{\tilde{M}}^{NStressG}\left(0,t\right)+\left(\xi_{1}+\frac{\lambda_{l}^{2}}{\lambda_{c}^{2}}\right)\;\frac{\partial {\tilde{M}}^{NStressG}\left(0,t\right)}{\partial \tilde{x}},$$

(42) $$-\frac{\partial^{3}\tilde{w}\left(1,t\right)}{\partial {\tilde{x}}^{3}}-\frac{1}{\lambda_{c}}\frac{\partial^{2}\tilde{w}\left(1,t\right)}{\partial {\tilde{x}}^{2}}=\;\;\frac{\xi_{1}}{\lambda_{c}}{\tilde{M}}^{NStressG}\left(1,t\right)+\;\left(\xi_{1}+\frac{\lambda_{l}^{2}}{\lambda_{c}^{2}}\right)\;\frac{\partial {\tilde{M}}^{NStressG}\left(1,t\right)}{\partial \tilde{x}}.$$

In addition, the bending moment in dimensionless form can be rewritten as:

(43) $${\tilde{M}}^{NStressG}\left(\tilde{x},t\right)=-\;\frac{\partial^{2}\tilde{w}\left(\tilde{x},t\right)\;}{\partial {\tilde{x}}^{2}}+\lambda_{c}^{2}\frac{\partial^{4}\tilde{w}\left(\tilde{x},t\right)\;}{\partial {\tilde{x}}^{4}}+\tilde{A_{\rho }}\left(\lambda_{c}^{2}\;\xi_{1}+\lambda_{l}^{2}\right)\left(\frac{\partial^{2}\tilde{w}\left(\tilde{x},t\right)}{\partial t^{2}}-{\tilde{g}}^{2}\frac{\partial^{4}\tilde{w}\left(\tilde{x},t\right)}{\partial {\tilde{x}}^{2}\partial t^{2}}\right)\;+\left(\lambda_{c}^{2}\;\xi_{1}+\lambda_{l}^{2}\right)\left(\left(\tilde{N^{T}}+\tilde{N^{C}}\right)\frac{\partial^{2}\tilde{w}\left(\tilde{x},t\right)}{\partial {\tilde{x}}^{2}}\right).$$

## 5. Solution Procedure

The natural frequencies and mode shapes of flexural vibrations are here evaluated by employing the classical separation of spatial and time variables:

(44) $$\tilde{w}\left(\tilde{x},t\right)=\tilde{W}\left(\tilde{x}\right)e^{i\tilde{\omega }t},$$

where $\tilde{\omega }$ denotes the dimensionless natural frequency of flexural vibrations. 

By substituting Equation (44) into Equations (38)–(43), the following dimensionless governing equations of the linear transverse free vibrations based on NStressG can be rewritten in terms of non-dimensional spatial shape $\tilde{W}\left(\tilde{x}\right)$ as:

- Dimensionless free vibration equation in terms of spatial shape

(45) $$\lambda_{c}^{2}\frac{\partial^{6}\tilde{W}\left(\tilde{x}\right)}{\partial {\tilde{x}}^{6}}+\frac{\partial^{4}\tilde{W}\left(\tilde{x}\right)}{\partial {\tilde{x}}^{4}}\left({\tilde{\omega }}^{2}\;\left(\lambda_{c}^{2}\;\xi_{1}+\lambda_{l}^{2}\right){\tilde{g}}^{2}+\left(\lambda_{c}^{2}\;\xi_{1}+\lambda_{l}^{2}\right)\left(\tilde{N^{T}}+\tilde{N^{C}}\right)-1\right)\;\;\;\;\;\;\;\;\;\;\;\;-\frac{\partial^{2}\tilde{W}\left(\tilde{x}\right)}{{\tilde{x}}^{2}}\left({\tilde{\omega }}^{2}\left(\lambda_{c}^{2}\xi_{1}+\lambda_{l}^{2}\right)+{\tilde{g}}^{2}{\tilde{\omega }}^{2}+\left(\tilde{N^{T}}+\tilde{N^{C}}\right)\right)+{\tilde{\omega }}^{2}\tilde{W}\left(\tilde{x}\right)=0;$$

- Dimensionless standard boundary conditions in terms of spatial shape

(46) $$\tilde{W}\left(\tilde{x}\right)=\tilde{W^{*}},\;\mathrm{or}\;\frac{\partial {\tilde{M}}^{NStressG}\left(\tilde{x}\right)}{\partial \tilde{x}}-\left(\tilde{N^{T}}+\tilde{N^{C}}\right)\frac{\partial \tilde{W}\left(\tilde{x}\right)}{\partial \tilde{x}}=\tilde{V},\;$$

(47) $$-\frac{\partial \tilde{W}\left(\tilde{x}\right)}{\partial \tilde{x}}=\frac{\partial {\tilde{W}}^{*}}{\partial \tilde{x}},\;\mathrm{or}\;{\tilde{M}}^{NStressG}\left(\tilde{x}\right)=\tilde{M};$$

- Dimensionless constitutive boundary conditions in terms of spatial shape

(48) $$-\frac{\partial^{3}\tilde{W}\left(0\right)}{\partial {\tilde{x}}^{3}}+\frac{1}{\lambda_{c}}\frac{\partial^{2}\tilde{W}\left(0\right)}{\partial {\tilde{x}}^{2}}=-\frac{\xi_{1}}{\lambda_{c}}{\tilde{M}}^{NStressG}\left(0\right)+\left(\xi_{1}+\frac{\lambda_{l}^{2}}{\lambda_{c}^{2}}\right)\frac{\partial {\tilde{M}}^{NStressG}\left(0\right)}{\partial \tilde{x}},$$

(49) $$-\frac{\partial^{3}\tilde{W}\left(1\right)}{\partial {\tilde{x}}^{3}}-\frac{1}{\lambda_{c}}\frac{\partial^{2}\tilde{W}\left(1\right)}{\partial {\tilde{x}}^{2}}=\frac{\xi_{1}}{\lambda_{c}}{\tilde{M}}^{NStressG}\left(1\right)+\left(\xi_{1}+\frac{\lambda_{l}^{2}}{\lambda_{c}^{2}}\right)\frac{\partial {\tilde{M}}^{NStressG}\left(1\right)}{\partial \tilde{x}};$$

- Dimensionless bending moment in terms of spatial shape

(50) $${\tilde{M}}^{NStressG}\left(\tilde{x}\right)=\lambda_{c}^{2}\frac{\partial^{4}\tilde{W}\left(\tilde{x}\right)\;}{\partial {\tilde{x}}^{4}}+\frac{\partial^{2}\tilde{W}\left(\tilde{x}\right)\;}{\partial {\tilde{x}}^{2}}\left({\tilde{\omega }}^{2}\left(\lambda_{c}^{2}\;\xi_{1}+\lambda_{l}^{2}\right){\tilde{g}}^{2}+\left(\lambda_{c}^{2}\;\xi_{1}+\lambda_{l}^{2}\right)\left(\tilde{N^{T}}+\tilde{N^{C}}\right)-1\right)-\;{\tilde{\omega }}^{2}\left(\lambda_{c}^{2}\;\xi_{1}+\lambda_{l}^{2}\right)\;\tilde{W}\left(\tilde{x}\right).$$

The analytical solution of Equation (45) can be expressed in the following form:

(51) $$\tilde{W}\left(\tilde{x}\right)=\sum_{k=1}^{6}q_{k}e^{\tilde{x}\;\beta_{k}},$$

wherein $\beta_{k}\;$are the roots of the characteristic equation, and $q_{k}\;$are six unknown constants to be determined by imposing suitable boundary conditions.

Note that, the six unknown constants can be obtained by satisfying boundary conditions Equations (46)–(49). Lastly, the linear fundamental natural frequencies of an FG nano-beam consists into solving the eigenvalue problem expressed in terms of a six dimensional array, $q=\left\{q_{1},\ldots ,q_{6}\right\}$. It can be noted that the corresponding characteristic equation is strongly nonlinear and is numerically solved by using a Wolfram language code written by the authors in Mathematica.

## 6. Results and Discussion

In this paragraph, a free vibration analysis of porous FG nano-beams under uniform hygro-thermal environment is carried out by considering two boundary conditions: clamped-free (C-F) and clamped-clamped (C-C). 

Firstly, the present approach has been validated by comparing the corresponding results, in terms of normalized frequency ratio between the dimensionless nonlocal fundamental frequency, $\tilde{\omega }$, and the dimensionless local natural frequency, ${\tilde{\omega }}_{loc}$, to those obtained by Barretta et al. in [45] assuming T=C=0. In particular, Table 3 and Table 4 collect the values of the aforementioned frequency ratio evaluated for two values of the mixture parameter (0.0, 0.5), varying $\lambda_{l}\;$and $\lambda_{c}$ in the sets {0.1, 0.3, 0.5} and {0+, 0.2, 0.4, 0.6, 0.8, 1.0}, respectively. It is worth noting that $\tilde{\omega }$ and ${\tilde{\omega }}_{loc}$ have been evaluated for a given value of the non-dimensional gyration radius, $\tilde{g}$, equal to 1/20.

### 6.1. Influence of Hygro-Thermal Loads

**Figure 2:**
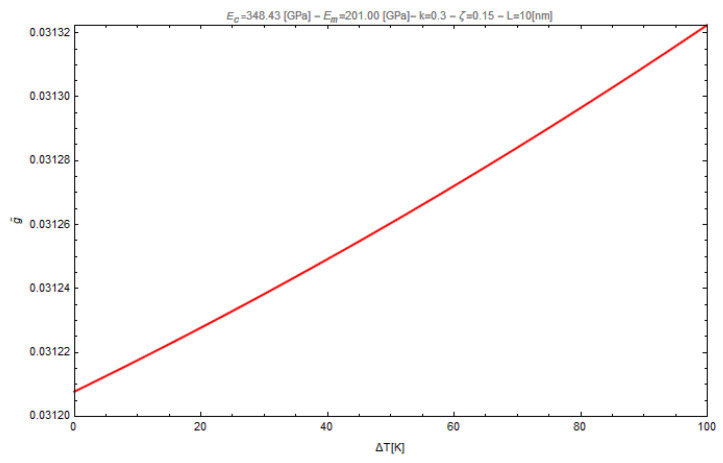
Influence of the temperature rise on the non-dimensional gyration radius.

In this subsection, the effects of hygro-thermal environment on the normalized fundamental flexural frequency of nano-beams, with length *L* = 10 nm and squared cross-section (*b* = *h* = 0.01 *L*), are presented by varying both the nonlocal parameter, $\lambda_{c}$, and the gradient length parameter, $\lambda_{l}$. In the case under investigation, the dimensionless nonlocal fundamental frequency, $\tilde{\omega }$, has been evaluated assuming *k* = 0.3 and *ζ* = 0.15 and ranging the temperature increment in the set {0, 25, 50, 75, 100 (K)} with a uniform and constant value of the moisture concentration equal to 2 (wt% H_2_O). For a better interpretation of the obtained results, it is interesting to show the influence of the temperature rise on the non-dimensional gyration radius of the nano-beam (Figure 2).

Note that the dimensionless local natural frequency, ${\tilde{\omega }}_{loc}$, has been obtained assuming *k* = 0.0, *ζ* = 0.0 (pure ceramic), T=C=0 and considering a value of the non-dimensional gyration radius, $\tilde{g}$, equal to zero.

From the numerical evidence of Table 5, Table 6, Table 7 and Table 8, it is possible to underline that the values of the normalized fundamental flexural frequency based on local/nonlocal stress-driven gradient theory of elasticity decrease as the temperature rise increases. Moreover, it is found that such values always increase by increasing the nonlocal parameter and by decreasing the gradient length parameter.

### 6.2. Influence of Gradient Index and Porosity Volume Fraction

In this subsection, the influences of the material gradient index, k, and of the porosity volume fraction, ζ on the frequency ratio between the nonlocal fundamental frequency, ω, of porous FG nano-beams and the corresponding local natural frequency, $\omega_{c,loc}$, of a purely nonporous ceramic nano-beam is presented. The values of *ω* and $\omega_{c,loc}$ have been evaluated assuming $\tilde{g}$ = 1/20 and two values of the mixture parameter, *ξ*_1_ = 0 and *ξ*_1_ = 0.5, and neglecting the hygro-thermal loadings.

**Figure 3:**
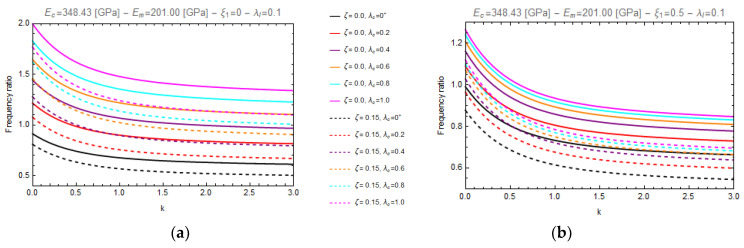
Effects of the gradient index (k) on the frequency ratio of FG cantilever nanobeam (C-F) for two different values of porosity volume fraction (*ζ* = 0.0, 0.15) and for two different values of mixture parameter: *ξ_1_ =* 0.0 (**a**) and *ξ_1_ =* 0.5 (**b**), varying nonlocal parameter, *λ_c_*, in the set {0+, 0.2, 0.4, 0.6, 0.8, 1.0}.

**Figure 4:**
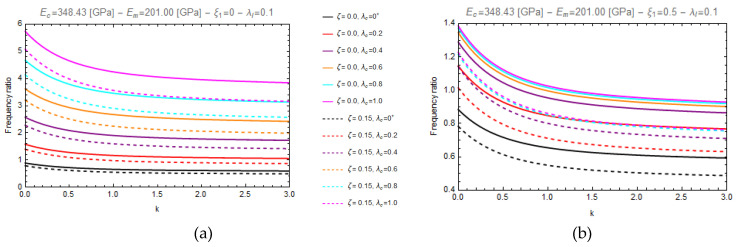
Effects of the gradient index (k) on the frequency ratio of FG fully clamped nanobeam (C-C) for two different values of porosity volume fraction (*ζ =* 0.0, 0.15) and for two different values of mixture parameter: *ξ_1_ =* 0.0 (**a**) and *ξ_1_ =* 0.5 (**b**), varying nonlocal parameter, *λ_c_*, in the set {0+, 0.2, 0.4, 0.6, 0.8, 1.0}.

**Figure 5:**
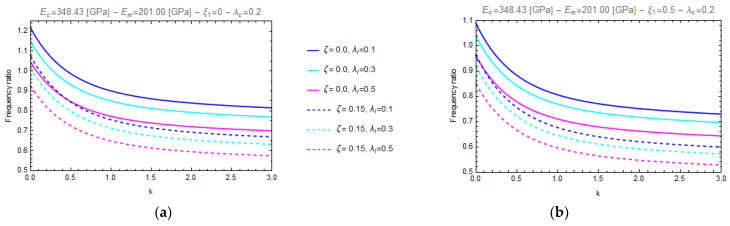
Effects of the gradient index (k) on the frequency ratio of FG cantilever nanobeam (C-F) for two different values of porosity volume fraction (ζ = 0.0, 0.15) and for two different values of mixture parameter: *ξ_1_ =* 0.0 (**a**) and *ξ_1_ =* 0.5 (**b**), varying the gradient length parameter, *λ_l_*, in the set {0.1, 0.3, 0.5}.

**Figure 6:**
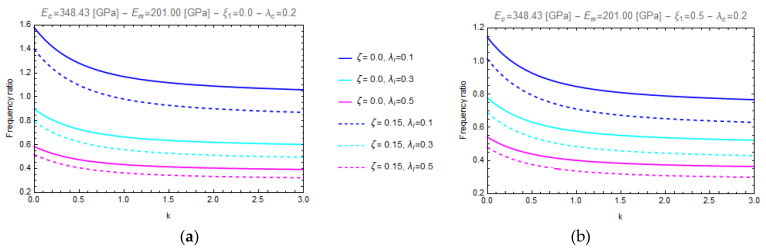
Effects of the gradient index (k) on the frequency ratio of FG fully clamped nanobeam (C-C) for two different values of porosity volume fraction (ζ = 0.0, 0.15) and for two different values of mixture parameter: *ξ_1_ =* 0.0 (**a**) and *ξ_1_ =* 0.5 (**b**), varying the gradient length parameter, *λ_l_*, in the set {0.1, 0.3, 0.5}.

In particular, Figure 3 and Figure 4 show the curves of the aforementioned frequency ratio versus the gradient index, assuming $\lambda_{l}=0.1,\;$with $\lambda_{c}$ ranging in the intervals [0.0^+^–1.0], while Figure 5 and Figure 6 plot the curves of the frequency ratio $\lambda_{l}\;$ranging in the set {0.1, 0.3, 0.5} for a given value of $\lambda_{c}$ equal to 0.2.

From these figures, it can be observed that all the frequency ratio curves tend to decrease as the material gradient index, *k*, increases and that the continuous lines, corresponding to nonporous FG nano-beams (*ζ* = 0.0), always present greater values than the dashed ones, which correspond to porous FG nano-beams (*ζ* = 0.15).

Moreover, on one hand (Figure 3 and Figure 4) it is found that an increase in the values of the nonlocal parameter, $\lambda_{c}$, causes an increase of the frequency ratio, but on the other hand (Figure 5 and Figure 6) it can be seen that as the gradient length parameter $\lambda_{l}\;$increases, the values of the frequency ratio decrease. Finally, one can find that the aforementioned frequency ratio decreases by increasing the mixture parameter.

**Figure 7:**
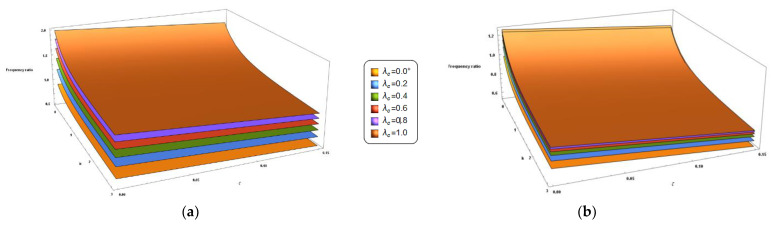
Cantilever porous FG nano-beam. 3D-plot of the frequency ratio in terms of the gradient index (k) and the porosity volume fraction (ζ) carrying the nonlocal parameter *λ_c_* in the set {0+, 0.2, 0.4, 0.6, 0.8, 1.0} and for two different values of the mixture parameter: *ξ_1_ =* 0.0 (**a**) and *ξ_1_ =* 0.5 (**b**).

**Figure 8:**
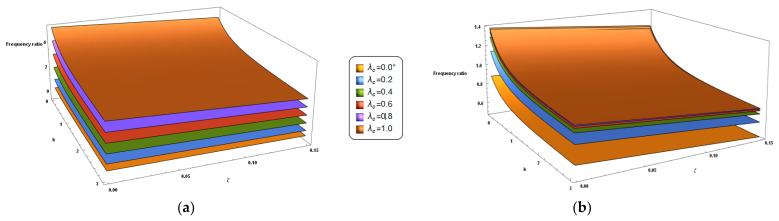
Fully clamped porous FG nano-beam. 3D-plot of the frequency ratio in terms of the gradient index (k) and the porosity volume fraction (ζ) carrying the nonlocal parameter *λ_c_* in the set {0+, 0.2, 0.4, 0.6, 0.8, 1.0} and for two different values of the mixture parameter: *ξ_1_ =* 0.0 (**a**) and *ξ_1_ =* 0.5 (**b**).

Finally, the coupled effect of *k* and ζ on the frequency ratio is shown in the 3D plots of Figure 7 and Figure 8 for the cantilever and the fully clamped nanobeam, respectively. From these figures it can be observed that the frequency ratio of the FG nano-beams under investigation increases by increasing the nonlocal parameter and decreases by decreasing the material gradient index and the porosity volume fraction. In addition, an increase in the values of the mixture parameter always results in a decrease of the frequency ratio.

## 7. Conclusions

The dynamic behavior of Bernoulli–Euler nano-beams made of a metal-ceramic functionally graded porous material subjected to hygro-thermal environments is examined in this manuscript. The governing equations are derived by employing Hamilton’s principle on the basis of the local/nonlocal stress gradient theory of elasticity (L/NStressG). The free vibration analysis is carried out by considering two different kinematic boundary conditions of engineering interest: Clamped-free (C-F) and clamped-clamped (C-C).

In particular, the effects of several parameters on both the thermo-elastic material properties and the structural response of the FG nano-beams, such as the porosity volume fraction and the material gradient index, the nonlocal parameter, the gradient length parameter and the mixture parameter, as well as the hygro-thermal loadings, have been investigated by using a Wolfram language code developed in Mathematica. Moreover, a comparison between the results of the present approach with those already available in current literature has been successfully presented.

The main outcomes of the present study may be summarized as follows:

- Influence of the porosity volume fraction and the gradient index*:* by increasing the gradient index and the porosity volume fraction, the axial and bending stiffnesses decrease, thus resulting in a decrease in the flexural frequency;
- Influence of the nonlocal parameter: the flexural frequency always increases with increasing the nonlocal parameter;
- Influence of the gradient length parameter: the flexural frequency always decreases by increasing the gradient length parameter;
- Influence of the mixture parameter: an increase in the values of the mixture parameter always results in a decrease of the flexural frequency;
- Influence of the hygro-thermal loadings: an increase of the temperature leads to an abatement of the thermo-elastic properties of the porous FG material and a decrease in the flexural frequency of the FG nano-beams due to a decrease in the axial and bending stiffnesses.

In conclusion, the proposed approach, based on L/NStressG plays an important role in revealing stiffness-hardening or stiffness-softening mechanical and dynamic behaviors in small-scaled structures, especially in temperature-dependent porous FG nano-beams.

## Figures and Tables

**Table 1:** Thermo-elastic properties of metal (SuS_3_O_4_) and ceramic (Si_3_N_4_).

Material	Properties	Unit	*P*_0_
Ceramic (Si_3_N_4_)	*E_c_*	(GPa)	348.40
	*ρ_c_*	(kg/m^3^)	2325
	*α_c_*	(K^−1^)	0.00000587
	*β_c_*	(wt% H_2_O)^−1^	0.0
Metal (SuS_3_O_4_)	*E_m_*	(GPa)	201.04
	*ρ_m_*	(kg/m^3^)	8011
	*α_m_*	(K^−1^)	0.00001233
	*β_m_*	(wt% H_2_O)^−1^	0.0005

**Table 2:** Coefficients of material phases for metal *(*SuS_3_O_4_) and ceramic (Si_3_N_4_).

		Ceramic (Si_3_N_4_)				Metal (SuS_3_O_4_)			
Coefficients	Unit	*E_c_*	*ρ* *_c_*	*α* *_c_*	*β* *_c_*	*E_m_*	*ρ* *_m_*	*α* *_m_*	*β* *_m_*
X_−1_	(K)	0	0	0	0	0	0	0	0
X_1_	(K^−1^)	−0.0003070	0	0.0009095	0	0.0003079	0	0.0008086	0
X_2_	(K^−2^)	2.160 × 10^−7^	0	0	0	−6.534 × 10^−7^	0	0	0
X_3_	(K^−3^)	−8.946 × 10^−11^	0	0	0	0	0	0	0

**Table 3:** Normalized fundamental flexural frequency of cantilever nano-beam (C-F) for T=C=0.

$\lambda_{c}$	$\frac{\tilde{\omega }}{{\tilde{\omega }}_{loc}}\;;$ T=C=0.											
	$\xi_{l}=0.0$						$\xi_{l}=0.5$					
	$\lambda_{l}=0.1$	Ref. [45]	$\lambda_{l}=0.3$	Ref. [45]	$\lambda_{l}=0.5$	Ref. [45]	$\lambda_{l}=0.1$	Ref. [45]	$\lambda_{l}=0.3$	Ref. [45]	$\lambda_{l}=0.5$	Ref. [45]
0+	0.99145	0.99145	0.92475	0.92475	0.82685	0.82685	0.99096	0.99096	0.92435	0.92435	0.82656	0.82656
0.2	1.21717	1.21717	1.14936	1.14936	1.04530	1.04530	1.08962	1.08962	1.04005	1.04005	0.96049	0.96049
0.4	1.44445	1.44445	1.38385	1.38385	1.28526	1.28526	1.16113	1.16113	1.12886	1.12886	1.07277	1.07277
0.6	1.64847	1.64847	1.59405	1.59405	1.50187	1.50187	1.20831	1.20831	1.18632	1.18632	1.12796	1.12796
0.8	1.83235	1.83235	1.78272	1.78272	1.69628	1.69628	1.24099	1.24099	1.22520	1.22520	1.16337	1.16337
1.0	2.00041	2.00041	1.95455	1.95455	1.87306	1.87306	1.26483	1.26483	1.25298	1.25298	1.18922	1.18922

**Table 4:** Normalized fundamental flexural frequency of fully clamped (C-C) nano-beam for T=C=0.

$\lambda_{c}$	$\frac{\tilde{\omega }}{{\tilde{\omega }}_{loc}};$ T=C=0.											
	$\xi_{1}=0.0$						$\xi_{1}=0.5$					
	$\lambda_{l}=0.1$	Ref. [45]	$\lambda_{l}=0.3$	Ref. [45]	$\lambda_{l}=0.5$	Ref. [45]	$\lambda_{l}=0.1$	Ref. [45]	$\lambda_{l}=0.3$	Ref. [45]	$\lambda_{l}=0.5$	Ref. [45]
0+	0.89165	0.89165	0.52522	0.52522	0.34619	0.34619	0.88416	0.88416	0.52314	0.52314	0.34529	0.34529
0.2	1.58127	1.58127	0.89822	0.89822	0.58545	0.58545	1.14531	1.14531	0.77938	0.77938	0.54126	0.54126
0.4	2.57577	2.57577	1.38724	1.38724	0.93713	0.93713	1.28946	1.28946	1.02374	1.02374	0.77625	0.77625
0.6	3.61940	3.61940	2.01640	2.01640	1.30727	1.30727	1.34633	1.34633	1.16750	1.16750	0.95453	0.95453
0.8	4.67784	4.67784	2.59796	2.59796	1.68291	1.68291	1.37237	1.37237	1.24944	1.24944	1.07846	1.07846
1.0	5.74258	5.74258	3.18308	3.18308	2.06089	2.06089	1.38608	1.38608	1.29819	1.29819	1.16320	1.16320

**Table 5:** Normalized fundamental flexural frequency of cantilever nano-beam (C-F) assuming $\xi_{1}=0.0$.

$\frac{\tilde{\omega }}{{\tilde{\omega }}_{loc}}\;$−$\;\xi_{1}=0.0\;$− ΔC=2.												
$\lambda_{c}$	ΔT=25			ΔT=50			ΔT=75			ΔT=100		
	$\lambda_{l}=0.1$	$\lambda_{l}=0.3$	$\lambda_{l}=0.5$	$\lambda_{l}=0.1$	$\lambda_{l}=0.3$	$\lambda_{l}=0.5$	$\lambda_{l}=0.1$	$\lambda_{l}=0.3$	$\lambda_{l}=0.5$	$\lambda_{l}=0.1$	$\lambda_{l}=0.3$	$\lambda_{l}=0.5$
0+	0.98544	0.91112	0.81096	0.98543	0.91061	0.81076	0.95774	0.89743	0.78232	0.89187	0.85324	0.71161
0.1	1.13066	1.06061	0.95190	1.07907	1.00592	0.89094	1.02226	0.94452	0.81943	0.95918	0.87469	0.73300
0.2	1.36641	1.30477	1.20421	1.32124	1.25788	1.15415	1.27247	1.20677	1.09847	1.21960	1.15076	1.03596
0.3	1.57748	1.52261	1.42958	1.53723	1.48128	1.38631	2.49424	1.43689	1.33931	1.44822	1.38908	1.28804
0.4	1.76687	1.71711	1.63040	1.73033	1.67981	1.59176	1.69154	1.64008	1.55031	1.65033	1.59771	1.50575
0.5	1.93929	1.89349	1.81207	1.90563	1.85925	1.77684	1.87005	1.82299	1.73933	1.83242	1.78454	1.69935

**Table 6:** Normalized fundamental flexural frequency of cantilever nano-beam (C-F) assuming $\xi_{1}=0.5$.

	$\frac{\tilde{\omega }}{{\tilde{\omega }}_{loc}}\;$−$\;\xi_{1}=0.5\;$− ΔC=2.											
$\lambda_{c}$	ΔT=25			ΔT=50			ΔT=75			ΔT=100		
	$\lambda_{l}=0.1$	$\lambda_{l}=0.3$	$\lambda_{l}=0.5$	$\lambda_{l}=0.1$	$\lambda_{l}=0.3$	$\lambda_{l}=0.5$	$\lambda_{l}=0.1$	$\lambda_{l}=0.3$	$\lambda_{l}=0.5$	$\lambda_{l}=0.1$	$\lambda_{l}=0.3$	$\lambda_{l}=0.5$
0+	0.96700	0.89741	0.79637	0.91465	0.85428	0.76812	0.85375	0.79756	0.71106	0.75051	0.71109	0.59731
0.1	1.00082	0.94827	0.86294	0.94694	0.89096	0.79867	0.88681	0.82572	0.72204	0.81887	0.75007	0.62700
0.2	1.07717	1.04295	0.98332	1.02690	0.99065	0.92713	1.07134	0.93237	0.86316	0.90968	0.86669	0.78904
0.3	1.12808	1.10478	1.06236	1.08041	1.05580	1.01090	1.02814	1.00180	0.95349	0.97042	0.94176	0.88701
0.4	1.16349	1.14676	1.11549	1.11764	1.10001	1.06701	1.06757	1.04878	1.01348	1.01256	0.99224	0.95388
0.5	1.18936	1.17682	1.15296	1.14484	1.13164	1.10651	1.09635	1.08231	1.05554	1.04325	1.02813	0.99921

**Table 7:** Normalized fundamental flexural frequency of fully clamped nano-beam (C-C) for $\xi_{1}=0.0$.

	$\frac{\tilde{\omega }}{{\tilde{\omega }}_{loc}}\;$−$\;\xi_{1}=0.0\;$− ΔC=2.											
$\lambda_{c}$	ΔT=25			ΔT=50			ΔT=75			ΔT=100		
	$\lambda_{l}=0.1$	$\lambda_{l}=0.3$	$\lambda_{l}=0.5$	$\lambda_{l}=0.1$	$\lambda_{l}=0.3$	$\lambda_{l}=0.5$	$\lambda_{l}=0.1$	$\lambda_{l}=0.3$	$\lambda_{l}=0.5$	$\lambda_{l}=0.1$	$\lambda_{l}=0.3$	$\lambda_{l}=0.5$
0+	0.87793	0.51401	0.34059	0.87788	0.51401	0.34059	0.87784	0.51358	0.34056	0.87775	0.51313	0.34050
0.1	1.56938	0.88682	0.57162	1.56718	0.88284	0.56538	1.56490	0.87869	0.55883	1.56254	0.87438	0.55194
0.2	2.55988	1.35548	0.92572	2.55854	1.35840	0.92207	2.55716	1.36141	0.91826	2.55572	1.29619	0.91430
0.3	2.59834	2.00256	1.29562	3.59739	2.00090	1.29307	3.59639	1.99917	1.29041	3.59536	1.99737	1.28765
0.4	4.65122	2.58140	1.67011	4.65046	2.58011	1.66815	4.64967	2.57877	1.66611	4.64886	2.57738	1.66399
0.5	5.71022	3.16352	2.04652	5.70959	3.16247	2.04492	5.70892	3.16137	2.04326	5.70824	3.16023	2.04154

**Table 8:** Normalized fundamental flexural frequency of fully clamped nano-beam (C-C) for $\xi_{1}=0.5$.

	$\frac{\tilde{\omega }}{{\tilde{\omega }}_{loc}}\;$−$\;\xi_{1}=0.5\;$− ΔC=2.											
$\lambda_{c}$	ΔT=25			ΔT=50			ΔT=75			ΔT=100		
	$\lambda_{l}=0.1$	$\lambda_{l}=0.3$	$\lambda_{l}=0.5$	$\lambda_{l}=0.1$	$\lambda_{l}=0.3$	$\lambda_{l}=0.5$	$\lambda_{l}=0.1$	$\lambda_{l}=0.3$	$\lambda_{l}=0.5$	$\lambda_{l}=0.1$	$\lambda_{l}=0.3$	$\lambda_{l}=0.5$
0+	0.86807	0.50998	0.34009	0.84925	0.49179	0.31288	0.84524	0.49166	0.30845	0.80905	0.48723	0.30840
0.1	1.13373	0.76745	0.52694	1.13089	0.76307	0.52041	1.12794	0.75850	0.51354	1.12487	0.75374	0.50631
0.2	1.27747	1.01222	0.76431	1.27501	1.00906	0.76006	1.27244	1.00576	0.75562	1.26987	1.00233	0.75100
0.3	1.33415	1.15579	0.94297	1.33180	1.15304	0.93960	1.32936	1.15020	0.93608	1.32682	1.14726	0.93243
0.4	1.36010	1.23753	1.06684	1.35780	1.23499	1.06389	1.35541	1.23235	1.06081	1.35293	1.22962	1.05762
0.5	1.37377	1.28614	1.15145	1.37149	1.28371	1.14873	1.36912	1.28118	1.14589	1.36667	1.27855	1.14295

## Data Availability

The data presented in this study are available on request from the corresponding author.
